# Supplementary material for: Development of Hydroxyapatite Coatings for Orthopaedic Implants from Colloidal Solutions: Part 2—Detailed Characterisation of the Coatings and Their Growth Mechanism
Source: Nanomaterials (Basel). 2023 Sep 21;13(18):2606. doi: 10.3390/nano13182606 (PMC10535467; doi:10.3390/nano13182606)
Supplement: Supplementary file 1 [file nanomaterials-13-02606-s001.zip › nanomaterials-2585104-supplementary.pdf]

Title

Development of hydroxyapatite coatings for orthopaedic implants from colloidal solutions, Part 1: effect of solution concentration and deposition kinetics.

Authors

Bríd Murphy[1,2] Jhonattan Baez[1,2] and Michael A. Morris[1,2]

1. AMBER Research Centre, CRANN Institute, Trinity College Dublin, Dublin 2, Ireland

2. School of Chemistry, Trinity College Dublin, Dublin 2, Ireland

Supplemental Material

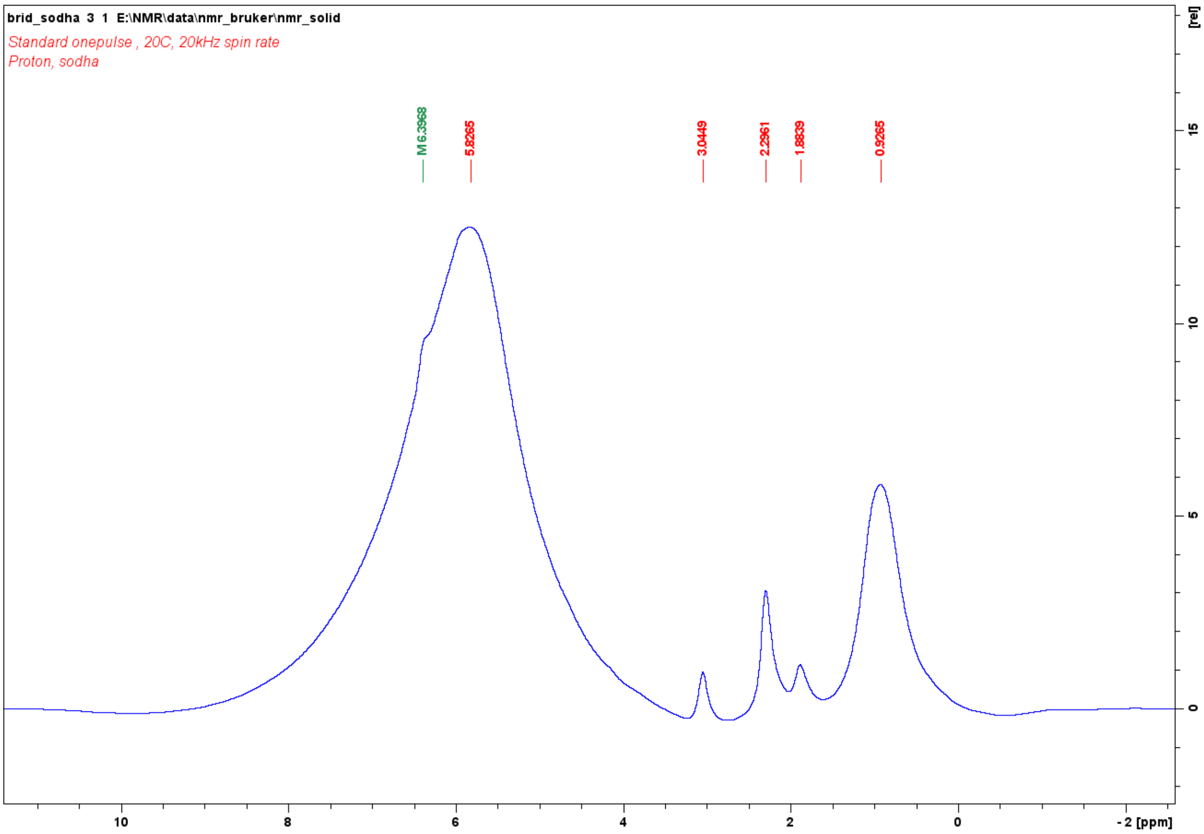

Figure 1: Solid state Nuclear Magnetic Resonance 1D ^{1}H proton spectrum from a standard one pulse sequence at 20kHz spin rate.

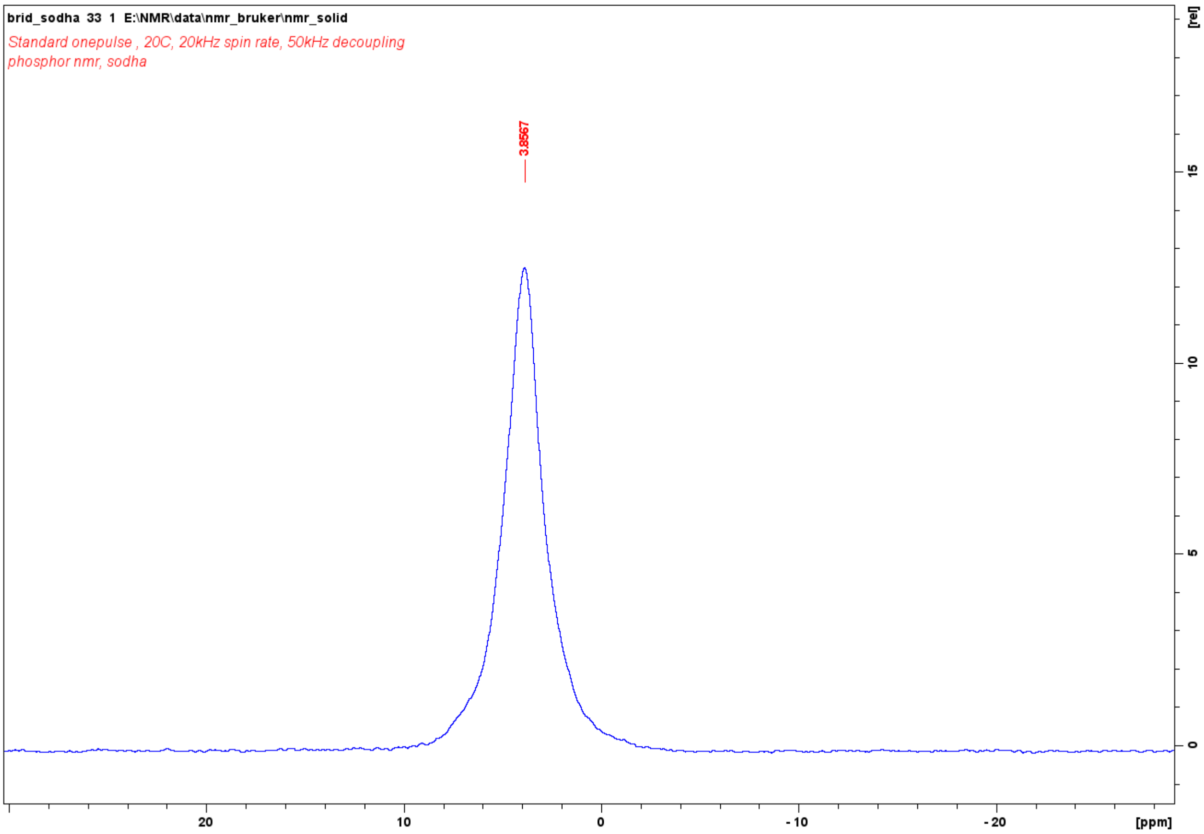

Figure 2: Solid state Nuclear Magnetic Resonance 1D ^{31}P (phosphorus) spectrum from a standard one pulse sequence at 20kHz spin rate with 50kHz decoupling.

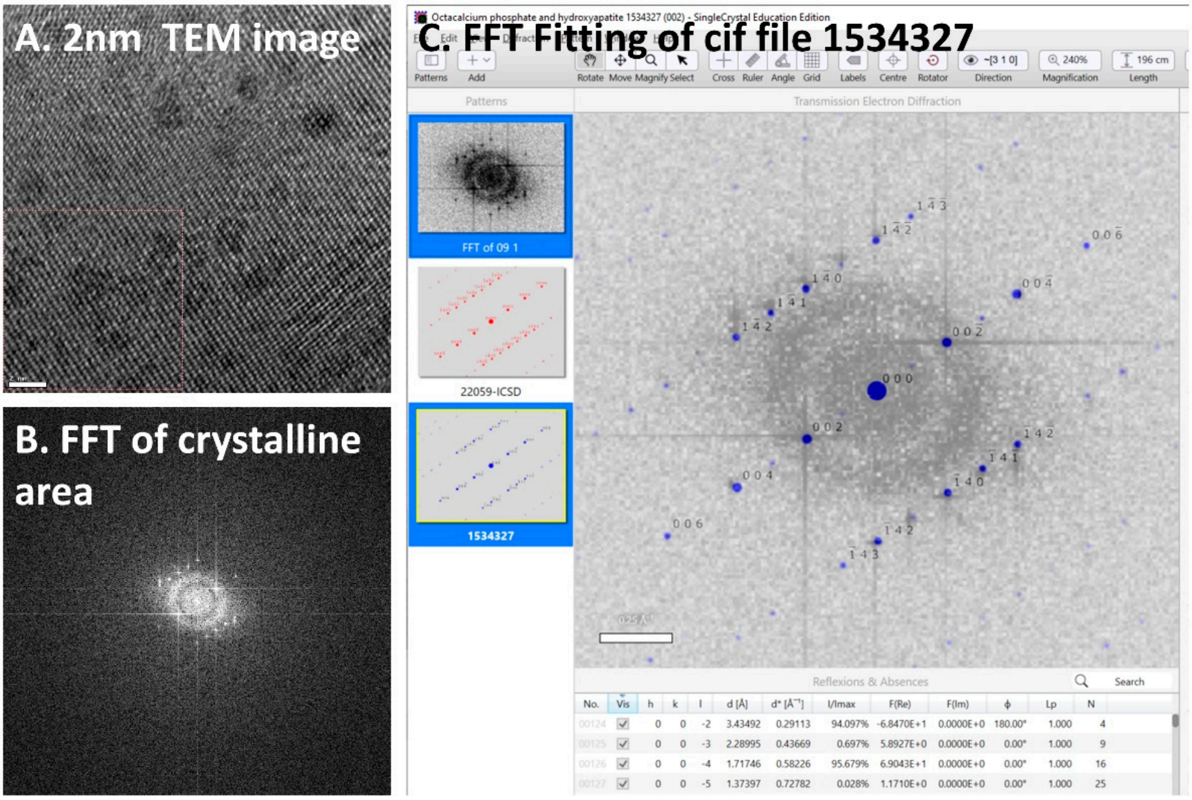

Figure 3: (A) high magnitude Transmission electron microscope (TEM) image of crystalline area within the deposited HA, insert box of one corner of highly symmetrical lattice fringes (B) Fast Fourier Transform measurement as applied to the highlighted square in A. and (C) Software interface from Single Crystal software (education edition) fitting the crystallographic information file (cif) number 1534327 to the FFT image in C.
